# From Injury to Innovation: Margaret Dix’s Pioneering Work in Neuro-Otology

**DOI:** 10.7759/cureus.73094

**Published:** 2024-11-05

**Authors:** L Stefano Ramirez-Gil, Cecilia Belen Espinosa-Arce

**Affiliations:** 1 Faculty of Medicine, Instituto Politecnico Nacional, Mexico City, MEX; 2 Otolaryngology - Head and Neck Surgery, Hospital Angeles Metropolitano, Mexico City, MEX

**Keywords:** biographies, historical vignette, historical vignettes, medical innovation, medical stories

## Abstract

Margaret Ruth Dix made groundbreaking contributions to neuro-otology, particularly in the study of vestibular disorders. Together with Charles Hallpike, Dix developed the Dix-Hallpike maneuver, a diagnostic technique that is still widely used today for benign paroxysmal positional vertigo (BPPV). Their research provided critical insights into BPPV, although they initially misidentified its cause, attributing it to otolithic disturbances instead of semicircular canal dysfunction. In addition to her work on BPPV, Dix made important contributions to caloric testing, Meniere's disease, and pure tone audiometry for young children, among other areas. Her efforts led to more effective diagnostic and educational methods. Despite some early misconceptions, the work of Dix and Hallpike remains a cornerstone of neuro-otology. The Dix-Hallpike maneuver continues to be essential for diagnosing BPPV, and their research has had a lasting impact on clinical practice and scientific knowledge in the field.

## Introduction and background

Margaret Ruth Dix was born in 1902. She studied at the Royal Free School of Medicine for Women, graduating in 1937 [1]. After graduation, she was appointed as a house surgeon under Douglas McLaggan, which marked the beginning of her promising medical career [1,2].

During the Blitz in 1940, Dix was severely injured in an air raid, resulting in significant facial disfigurement. Despite undergoing multiple plastic and ophthalmic surgeries, the damage to her eye prevented her from continuing her surgical career. This traumatic experience, along with her mother's deafness, likely influenced her deep commitment to studying auditory and vestibular pathologies [1,2].

In 1945, she joined the Otological Research Unit at the National Hospital, Queen Square, London, where she conducted an investigation on deafness in ex-servicemen under the direction of Dr. C. S. Hallpike. This was the beginning of her career in neuro-otology [1,2].

Throughout her career, Dix published numerous scientific papers on auditory, vestibular, and neurological conditions. Her work earned her widespread recognition and numerous awards, including the W. J. Harrison Prize in Otology in 1954, the R. S. M. Dalby Prize in 1958, and the Norman Gamble Research Prize in 1980. In 1965, she was appointed honorary consultant to the National Hospital, a position she held until her retirement in 1976 at the age of 70. She was also an honorary member of the Barany Society and a member of the prestigious international Collegium Oto-Rhino-Laryngologicum Amicitiae Sacrum [2-3].

Margaret Dix passed away on December 9, 1991, at the age of 89 [1].

## Review

**Figure 1:**
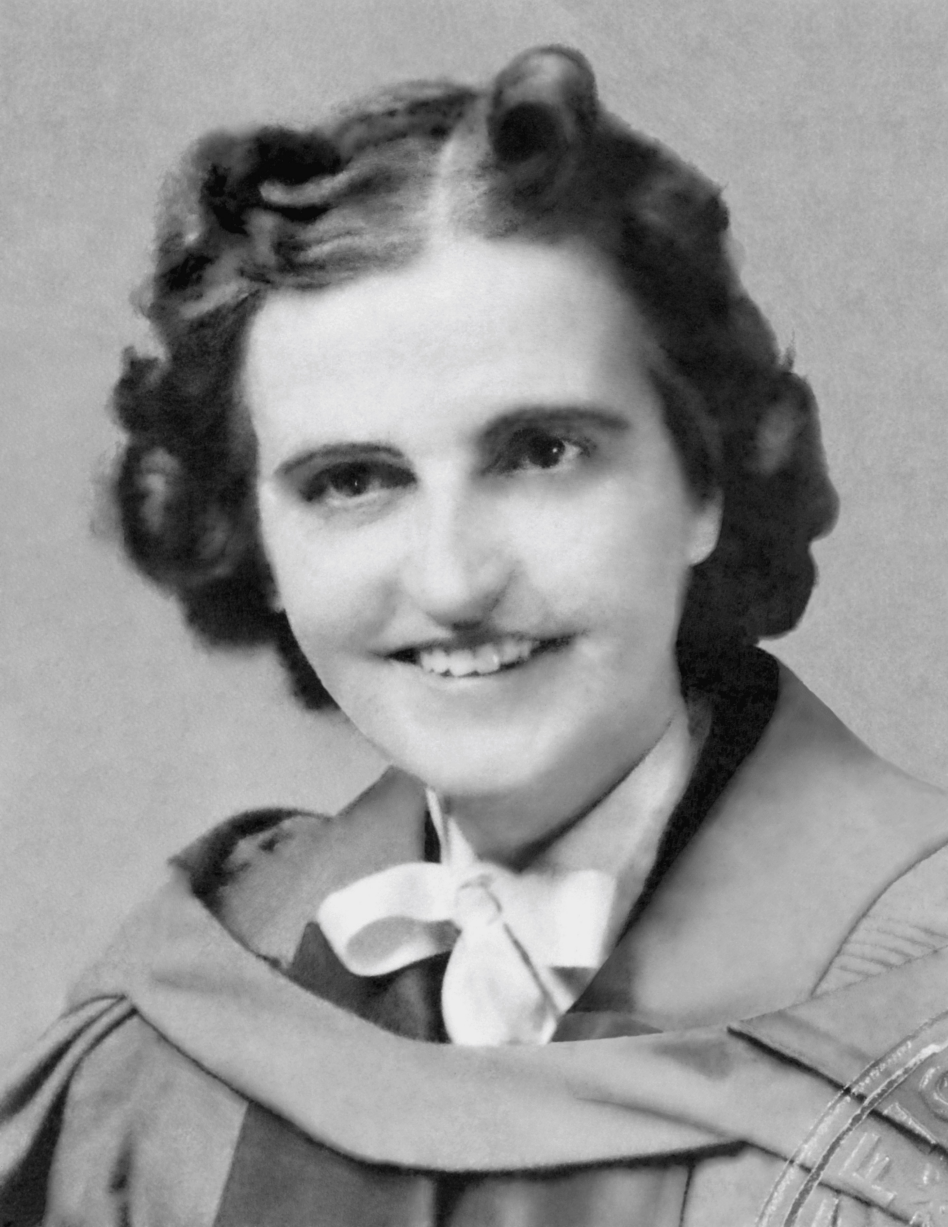
Margaret Ruth Dix Image owned by the Queen Square Archives [7]. Reproduced with permission. All rights reserved.

​​​​Margaret Dix (Figure 1) and Charles Hallpike's contributions to otology and neuro-otology are unparalleled, and their coauthored literature has left an indelible mark on these fields [2,4-6]. Among their many works, the 1952 publication titled "The Pathology, Symptomatology and Diagnosis of Certain Common Disorders of the Vestibular System" stands out as a cornerstone in contemporary neuro-otology. This seminal paper provided a comprehensive analysis of the three most common peripheral vertigo disorders: Ménière's disease, vestibular neuritis, and benign paroxysmal positional vertigo (BPPV) [6].

BPPV 

In 1921, Barany observed some remarkable clinical phenomena that seemed to result from a dysfunction in the otolith apparatus of the human ear. He described a patient who experienced severe vertigo and nystagmus when their head was in a specific position - lying on their back with the head turned to one side. The nystagmus was rapid and had a unique, rotatory pattern with a horizontal movement toward the ear that was closest to the ground [8]. Barany showed that it was not the movement of the head but its position in space that triggered these symptoms, leading him to conclude that the issue was related to a disorder in the otolith apparatus. He also noted that the nystagmus and vertigo would quickly disappear if the patient kept their head in the critical position. Importantly, there were no other signs of ear disease in Barany's patient; both hearing and caloric responses were normal, and neurological examination revealed no abnormalities [8,9].

Later, Dix and Hallpike expanded on Barany's work to highlight the sudden and episodic nature of the nystagmus and the generally benign course of the condition. They also developed a technique for reliably eliciting this type of positional vertigo [6,9].

**Figure 2:**
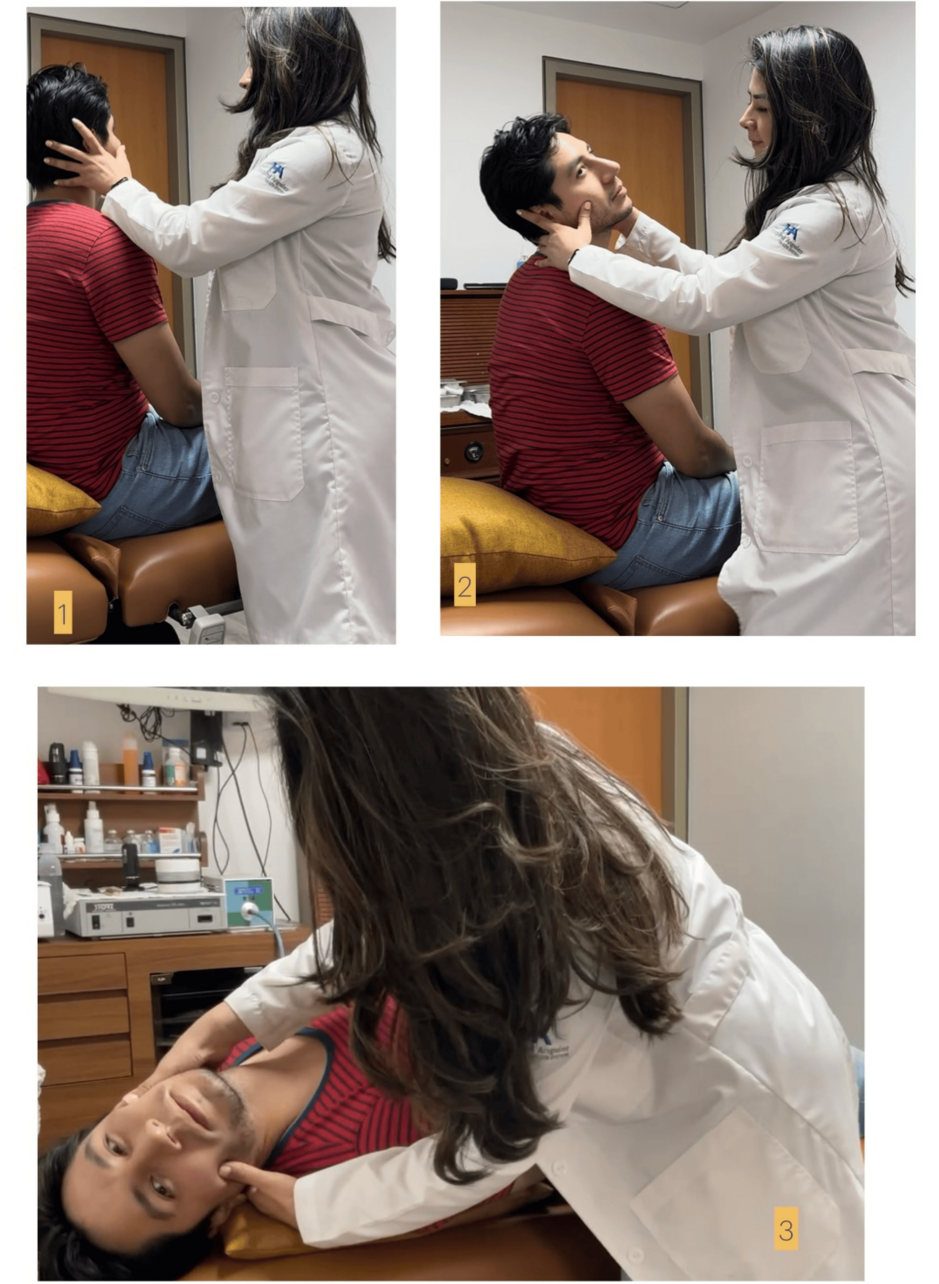
Dix-Hallpike Maneuver The individuals in these photos are the authors of this paper, and we consent to having our images included in this publication.

“The patient is laid supine upon a couch with his head just over its end. The head is then lowered some 30 degrees below the level of the couch and turned some 30 degrees to 45 degrees to one side. In taking up this position, the patient is first seated on the couch with the head turned to one side and the gaze fixed upon the examiner’s forehead. The examiner then grasps the patient’s head firmly between his hands and briskly pushes the patient back into the critical position” (Figure 2) [6].

This marked the first clear description of the provocative positioning technique, which is now the gold standard for diagnosing BPPV. Dix and Hallpike not only introduced this technique but also coined the term "positional vertigo of the benign paroxysmal type." Through their clinical evaluation of 100 cases, they identified the key symptoms of BPPV elicited by their maneuver: a delay in the onset of symptoms after positioning, a rapid increase in the severity of nystagmus and vertigo to a peak, linear-rotatory nystagmus with the fast phase beating toward the ear that is closest to the ground, a brief duration of nystagmus even when the offending head position is maintained, a reversal of vertigo and nystagmus to a milder form when the patient returns to a seated position, and fatigability, where symptoms lessen or disappear with repeated maneuvers [6,9].

The only error in their clinical description was that they characterized the linear component of the nystagmus as horizontal when it is actually primarily upbeat, especially when the gaze is directed away from the lower ear, except in rare cases of horizontal BPPV [9]. However, Dix and Hallpike incorrectly attributed BPPV to an otolithic disturbance [9].

Dix continued to believe that BPPV was caused by otolithic pathology [8] until the condition was later localized to dysfunction of the posterior semicircular canal [9,10-16]. Despite their substantial contributions, both Dix and Hallpike did not fully grasp the underlying pathophysiology of BPPV. They also did not recognize that the positioning techniques they employed were revealing pathology in the semicircular canals rather than in the utricle [8]. It is now known that in BPPV, the posterior canal is affected in 85% to 95% of cases, while the horizontal canal is involved in 5% to 15% of cases [17]. Anterior canal BPPV is extremely rare [18].

The Dix-Hallpike maneuver, while widely recognized for its diagnostic utility, has limitations. It is contraindicated in patients with conditions such as cervical instability, Arnold-Chiari malformation, and vertebrobasilar insufficiency, among others [17]. The maneuver's sensitivity and specificity can also vary depending on the clinician's expertise, with higher accuracy generally seen among specialists (sensitivity of 82% and specificity of 71%) [18].

Caloric test

In their 1949 research on caloric testing, Dix, Hallpike, and Harrison studied human subjects with severe streptomycin intoxication. They found that even when caloric responses were absent or greatly reduced, the optokinetic responses remained normal. This finding provided evidence that the reflex pathways responsible for optokinetic nystagmus operate independently of the vestibular nuclei [19].

Further investigations by Carmichael, Dix, and Hallpike revealed that the cortical areas involved in controlling the direction of caloric nystagmus are located in the posterior part of the temporal lobes. These areas are believed to regulate caloric nystagmus in specific directions by influencing the reflex reactivity of the vestibular nuclei [19]. Their 1954 findings also supported earlier theories proposed by Stenvers and others, which suggested that certain cortical areas are similarly involved in the directional control of optokinetic nystagmus [19].

Audiometry in children 

Dix and Hallpike conducted research on pure-tone audiometry in children under six years old to differentiate between deafness and intellectual and developmental disabilities, ensuring that appropriate interventions could be provided. One of the main challenges in administering speech and pure tone audiometry was gaining the cooperation of the young patients, as well as overcoming language limitations and effectively explaining the instructions. To address these issues, they developed the "peep show," a test similar to modern play audiometry. This test, which lasted 10 to 15 minutes, allowed them to obtain reliable threshold curves in deaf children of average intelligence as young as three years old. The equipment they used was simple, robust, and easy to operate, making the test both quick and effective [5]. They studied the test's effectiveness in 350 cases and published their findings, detailing how they managed to test restless, shy, or uncooperative children, including those with mental or motor disorders. Their general findings contributed to recommendations for special education for these patients [4].

## Conclusions

Margaret Dix and Charles Hallpike made pivotal contributions to our understanding of vestibular disorders, particularly BPPV. Their clinical work established foundational knowledge in the diagnosis and management of vestibular conditions. Despite some inaccuracies in their pathophysiological interpretations, their insights remain influential. The Dix-Hallpike maneuver, which bears Dix's name as the first author, continues to be a cornerstone in diagnosing BPPV. Their research has had a lasting impact, and their legacy is reflected in the ongoing relevance of their findings in contemporary neuro-otology.
